# The Role of Matrix Metalloproteinases in Periodontal Disease

**DOI:** 10.3390/ijerph17144923

**Published:** 2020-07-08

**Authors:** Vittorio Checchi, Tatjana Maravic, Pierantonio Bellini, Luigi Generali, Ugo Consolo, Lorenzo Breschi, Annalisa Mazzoni

**Affiliations:** 1 Unit of Dentistry and Oral-Maxillo-Facial Surgery, Department of Surgery, Medicine, Dentistry and Morphological Sciences with Transplant Surgery, Oncology and Regenerative Medicine Relevance, University of Modena and Reggio Emilia, 41125 Modena, Italy; pierantonio.bellini@unimore.it (P.B.); luigi.generali@unimore.it (L.G.); ugo.consolo@unimore.it (U.C.); 2 DIBINEM, University of Bologna, 40100 Bologna, Italy; tatjana.maravic2@unibo.it (T.M.); lorenzo.breschi@unibo.it (L.B.); annalisa.mazzoni@unibo.it (A.M.)

**Keywords:** matrix metalloproteinases, MMP, collagenases, periodontal disease

## Abstract

This review provides a detailed description of matrix metalloproteinases (MMPs), focusing on those that are known to have critical roles in bone and periodontal disease. Periodontal disease is an inflammatory process initiated by anaerobic bacteria, which promote the host immune response in the form of a complex network of molecular pathways involving proinflammatory mediators such as cytokines, growth factors, and MMPs. MMPs are a family of 23 endopeptidases, collectively capable of degrading virtually all extracellular matrix (ECM) components. This study critically discusses the available research concerning the involvement of the MMPs in periodontal disease development and progression and presents possible therapeutic strategies. MMPs participate in morphogenesis, physiological tissue turnover, and pathological tissue destruction. Alterations in the regulation of MMP activity are implicated in the manifestation of oral diseases, and MMPs comprise the most important pathway in tissue destruction associated with periodontal disease. MMPs can be considered a risk factor for periodontal disease, and measurements of MMP levels may be useful markers for early detection of periodontitis and as a tool to assess prognostic follow-ups. Detection and inhibition of MMPs could, therefore, be useful in periodontal disease prevention or be an essential part of periodontal disease therapy, which, considering the huge incidence of the disease, may greatly improve oral health globally.

## 1. Introduction

Periodontitis is an inflammatory disorder that causes tissue and bone loss as a consequence of various interactions between the host immune response and pathogenic bacteria [1]. This inflammatory process is originated by plaque biofilm that causes the loss of periodontal attachment, resulting, in the most severe cases, in tooth loss [2,3,4]. Though anaerobic bacteria are considered the starting agents, the disease progression is induced by the host response, which can be influenced by environmental and behavioral factors [5]. The disease development involves various interacting molecular pathways made of proinflammatory mediators such as growth factors, cytokines, matrix metalloproteinases (MMPs), and their inhibitors and regulators [6].

Several pieces of evidence suggest that MMPs comprise the most important pathway in tissue destruction associated with periodontal disease due to their role in the pathological breakdown of extracellular matrix (ECM) within periodontal tissues. The MMPs and their tissue inhibitors (TIMPs) have a key role in the physiological tissue remodeling of the periodontal tissues. However, disruption of the balance between MMPs and TIMPs has been observed in association with periodontal disease and tissue breakdown [7]. Pathogens in dental plaque are able to stimulate host cells to increase their MMP release, which is considered as one of the indirect mechanisms of tissue destruction occurring in periodontitis [8,9].

The aim of this review is to provide a detailed description of MMPs and of their critical role in bone and periodontal disease, discussing current knowledge regarding MMPs within the periodontal tissues in an attempt to better understand their role, nature, and functions in the extracellular matrix environment.

## 2. Matrix Metalloproteinases (MMPs)

MMPs, also called matrixins, are endogenous Zn^2+^- and Ca^2+^-dependent enzymes. These enzymes can degrade almost all ECM components, and thus play important roles in many biological and pathological processes. The understanding of the biochemical and structural properties of MMPs, including their catalytic mechanisms, activation, and substrate specificity, has improved considerably [10].

The MMP family has 23 members in humans, classified into six groups based on substrate specificity and homology: collagenases, gelatinases, stromelysins, matrilysins, membrane-type MMPs (MT-MMPs), and other MMPs (Table 1) [10,11,12].

Although this classification is practical in many cases, most MMPs can degrade several substrates with different specificities. For example, gelatinases can degrade several collagen types, especially type IV, and collagenases-1 and -3 (MMPs-1 and -13) also degrade gelatin, although the rate of this degradation is much slower than that of gelatinases (MMPs-2 and -9) [11]. For this reason, the molecular structure is often used to classify MMPs.

MMPs have prodomains and catalytic domains, as well as other domains governing factors such as substrate specificity, recognition, and interaction [10,13]. Like other proteolytic enzymes, MMPs are synthesized as inactive proenzymes or zymogens. The opening of the cysteine–zinc switch by proteolytic removal of the propeptide domain or ectopic disruption of the cysteine–zinc interaction is required for the activation of the MMP proenzymes [10,11]. The carboxyl terminus of the prodomain contains the thiol group of an unpaired, conserved cysteine, which maintains enzyme latency. At the active site, this cysteine serves as a fourth ligand that inactivates the catalytic zinc atom and excludes water. Disruption of the cysteine–zinc pair by proteolysis or a conformational change, resulting in the replacement of the thiol group by water, causes the activation process. The activation process is completed by the “cysteine switch” mechanism, in which the enzyme hydrolyzes the propeptide [14].

Collagenases, gelatinases, and MT-MMPs are directly involved in native fibrillar collagen degradation. MMPs induce triple helix unwinding, at least at the local level, enabling complete collagenolysis and the formation of 1/4- and 3/4-length fragments [15,16,17]. Interstitial collagens consist of three α chains with approximately 1000 residues and repeating Gly-X-Y triplets, in which X and Y are often proline and hydroxyproline. This triple helix composition renders interstitial collagens resistant to most proteinases, although collagenases and cathepsin K can cleave the helical structure [10,18]. However, before the collagenases can approach the cleavage site on the collagen molecule, C-telopeptides need to be removed by the telopeptidases (MMPs-2 and -9). This facilitates the unwinding of the triple helix and enables the cleavage by true collagenases [19,20]. After the collagen molecules are cleaved into 3/4- and 1/4-length fragments, they denature at body temperature and undergo degradation by nonspecific tissue proteinases and gelatinases [16,17].

Regulation of MMP activity can occur at multiple levels, including transcription, zymogen secretion, degranulation of intracellular-granule enzymes, localization in or outside the cell, internalization, extracellular inhibition, and clearance from the extracellular space [11]. TIMPs are involved in the local control of MMP activities in tissues. Whereas most TIMPs inhibit active MMPs, some also prevent proMMP activation; TIMP-1, for instance, binds to proMMP-9, and TIMPs-2 and -4 bind to proMMP-2. Besides inhibiting MMP activity, TIMPs also participate in several regulatory processes, such as embryogenesis, regulation of cell characteristics and processes (such as growth, survival, morphology, and apoptosis), inhibition of angiogenesis, and steroidogenesis [21,22,23,24]. Whereas TIMPs are the primary inhibitors of MMPs in tissues, α2-macroglobulin is the primary regulator of MMP activity in body fluids. This macroglobulin protein, abundant in plasma, is a general endopeptidase inhibitor that acts via enzyme entrapment and steric interference in large MMP substrate interactions, resulting in endocytosis of the α2-macroglobulin–MMP complex [10,25]. This process terminally eliminates the complex, rendering α2-macroglobulin important in irreversible MMP clearance; in contrast, TIMP-related MMP inhibition is reversible [11].

Numerous synthetic MMP inhibitors have been developed, and the number of these products continues to increase. Most of these inhibitors chelate or replace the zinc ion at the active site and/or interact with the MMP propeptide fragment; some may prevent MMP access and inhibit activity by coating the substrate [21].

## 3. The Role of MMPs in Periodontal Disease

**Figure 1:**
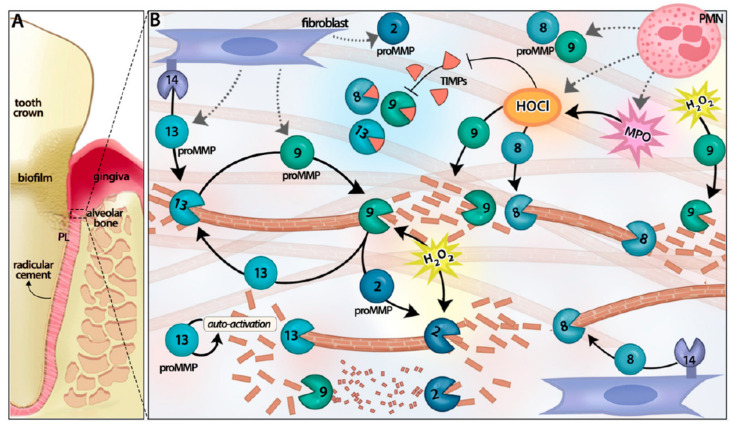
Matrix metalloproteinase (MMP) activation cascades in connective tissue catabolism during periodontitis. Full and partial circles with their respective number represent latent and activated specific MMPs. Brown T bars represent collagen fibers. (**A**) Tooth and its supporting structures: radicular cement, periodontal ligament (PL), and alveolar bone. (**B**) Membrane-bounded MMP-14 activates proMMP-13 to degrade type I collagen, which constitutes the bulk component of radicular cement, periodontal ligament (PL), and alveolar bone extracellular matrix; MMP-13 activates proMMP-9, which in turn might activate proMMP-2 and proMMP-13. MMP-2 and MMP-9 further process the gelatin resulting from collagenase activity. MMP-13 can undergo autoactivation by selfproteolysis. Reactive oxygen species, such as HOCl and H_2_O_2_, from phagocytes, can also modify the proteases/antiproteases balance, activating latent MMPs and inactivating the tissue inhibitor of MMP (TIMP)-1. MPO, myeloperoxidase. Image is taken from Franco et al. [8]; Reproduced with permission from Marcela Hernandez.

Although a number of MMPs have been described in gingival tissues (such as MMP-2, MMP-7, and MMP-14) [26], the most widely reported MMPs in gingival crevicular fluid (GCF) are MMP-8, MMP-9, and MMP-13 [27,28,29,30], and among the three collagenases (MMP-1, MMP-8, and MMP-13), MMP-8 accounts for 80% of the total collagenase protein found in the GCF, with smaller relative amounts of MMP-13 and MMP-1 in chronic periodontitis [31]. In particular, the active MMP-8 (aMMP-8, collagenase-2) derived from neutrophils is known to be the main host-cell-derived collagenase that leads to periodontal tissue destruction as a result of the degradation of gingival and periodontal ligament collagen [32,33] (Figure 1, [8]).

When comparing chronic periodontitis patients with gingivitis patients and healthy control subjects, higher levels of MMP-8 in saliva and higher MMP-9 levels in gingival crevicular fluid (GCF) were detected [34]. GCF MMP-8 activation was found to be the highest in chronic periodontitis patients compared with healthy subjects and gingivitis patients [35,36]. Recently, differentiating periodontitis patients from the controls, Gursoy et al. confirmed that salivary TIMP-1 and MMP-8 and -9 are increased in chronic periodontitis patients [37].

Apart from MMPs-8 and -9, other MMPs can also play a significant role in periodontal disease. The concentrations of MMP-3 and MMP-7 are markedly increased in the GCF of patients with periodontitis [36]. In cross-sectional studies, elevated GCF levels of MMP-7, TIMP-1 [38], laminin-5 gamma2-chain [39], and MMPs-25 and -26 [40] were reported in chronic periodontitis patients compared to gingivitis patients and healthy control subjects. 

GCF levels of MMPs in patients with periodontitis were also studied in terms of their diagnostic and prognostic values. A cross-sectional study by Ramseier et al. reported that salivary concentrations of MMPs-8, -9, and orthopantomograms combined with red complex bacteria can predict periodontal disease [41]. GCF levels of MMPs-9 and -13 were suggested as useful biomarkers for periodontitis progression when active sites were defined in moderate to advanced chronic periodontitis patients followed for 2 months [42,43]. Another cross-sectional study reported that GCF MMPs-8 and -9 levels are correlated with disease activity in chronic periodontitis patients [28]. Interesting data came from a clinical study on 28 chronic periodontitis patients and 22 controls [44]. The authors reported higher MMPs-3, -8, -9, and gelatinolytic activities in plasma of chronic periodontitis patients that decreased significantly 3 months after nonsurgical periodontal treatment. Kinane et al. [45] also reported that GCF MMP-8 levels decreased significantly 3 months after nonsurgical periodontal therapy in 20 chronic periodontitis patients. Persistent elevation of MMP-8 in GCF samples is regarded as indicating high risk and poor response to periodontal therapy [46]. Moreover, significant positive correlations were detected between GCF MMPs-8 and -9 activities and periodontal disease severity, together with negative correlations with TIMPs-1 and -2 levels [47]. Furthermore, a chair-side MMP-8 test was indicated to effectively differentiate clinically healthy sites and gingivitis from chronic periodontitis and also effectively monitor the treatment of chronic periodontitis patients [48].

As for bone resorption, MMP-9 is likely the most important proteinase involved in this process, as osteoclasts express this enzyme at a tremendously high level [49,50]. However, there are contradictory reports about the specific role of MMP-9 in bone resorption. For example, certain authors have suggested that MMPs make, at best, a very small contribution to the bone-resorptive activity of osteoclasts [51] and that the selective inhibitor of MMP-9, TIMP-1, did not show a significant inhibitory effect on osteoclastic bone resorption [52], while other studies have reported that MMP-9 might play an essential role in the bone resorption caused by osteoclasts [53] and that patients with MMP-9 genotypes, in association with their soluble protein, may have an increased risk of developing chronic periodontitis [54].

As seen from the previous studies, various proteolytic enzymes, like MMPs and elastases, at the end of the inflammatory cascade, can actively destroy periodontal tissues (Table 2).

It is noteworthy that only the active form of these enzymes is catalytically capable of acting in a destructive manner [32]. A specific therapy focused on the inhibition of MMP activities could be useful as a potential therapeutic strategy towards periodontitis treatment. This, in addition to clinical treatments such as scaling and root planing, would improve disease prognosis. Previous studies have also shown that MMPs are present in peri-implant sulcular fluid and can play a pathologic role in peri-implant bone loss [55,56]. MMP-8 has been reported to be present in peri-implant sulcular fluid. The polymorphism in the promoter of the MMP-8 gene (C-799T) could be a risk factor for early implant failure. This polymorphism could be used as a genetic marker for unsuccessful implants. In addition, the polymorphism in the promoter region of the MMP-1 gene (guanine inserted at position -1607) seemed to be strongly associated with early implant failure in a sample of 180 nonsmoking patients attending two dental clinics in Brazil [57]. Perhaps the discovery of several genetic markers related with early implant failure could be of clinical value for the precise and early identification of individuals at high risk for implant loss. It could lead to a more strict selection of patients, and in the future, individual therapeutics could be developed, thereby increasing implant success rates [57]. Therefore, the presence of MMPs that could contain these genetic markers could be extremely indicative of a patient with a high risk of implant failure.

## 4. MMP Inhibitors

In the late 1990s, macromolecular inhibitors (monoclonal antibodies and natural TIMPs) and small molecules (natural and synthetic) were already considered potential treatments for diseases with an excess of MMP activity [58].

In the last two decades, the development of MMP inhibitors that can act selectively under pathological conditions has been pursued intensively by the pharmaceutical industry, given the associations of MMPs with numerous diseases, such as cancer, arthritis, and conditions related to tissue remodeling [59]. However, few drugs with these properties have been found to effectively inhibit individual MMPs [60]. Furthermore, the toxicity of systemically delivered MMP inhibitors that were designed to treat specific pathological conditions, and their uncontrolled actions on target and antitarget members of the metzincin family, represent important challenges in efforts to develop synthetic products [61]. Clinical trials have shown that broad-spectrum MMP inhibitors lack specificity, have poor pharmacokinetics, and inhibit closely related enzymes [62]. All MMPs and the related enzymes share a highly homologous catalytic domain, which has increased the difficulty of specific-antagonist drug candidate development [63] but provides an advantage in the development of broad-spectrum inhibitors for dentin-matrix-bound and salivary MMPs.

Targeted therapy focused on MMP activity inhibition could be indicated as an ideal therapeutic option in the treatment of periodontal diseases. In addition to periodontal therapy, such as scaling and root planing and osseous surgery, this therapeutic strategy could be useful in improving the prognosis of periodontal infection.

Among other molecules, MMP inhibitors adopted in periodontal disease therapy are modified tetracyclines. Tetracyclines are antibiotics able to inhibit the breakdown of connective tissue. Inhibitors have been obtained through chemical modification of molecules from the tetracycline family, after the separation of antibiotic and protease inhibitory activities [58].

Minocycline provided the first evidence of MMP inhibition by tetracycline (TC) and its derivatives in the oral environment, as it was observed to inhibit the collagenolytic activity of gingival crevicular fluid in the absence of bacteria [64,65]. TCs are antibiotics with cationic chelating properties that inhibit MMP activity in the extracellular environment [66] and prevent proMMP activation through oxidation by scavenging reactive oxygen species [67]. TCs downregulate transcriptional MMP levels at the intracellular level [68,69,70]. Doxycycline hyclate is a low-dose tetracycline analog deprived of antimicrobial activity and is indicated for the treatment of periodontal diseases, acting through the inhibition of MMP-8 and MMP-13 protease mechanisms. The therapeutic effect of this antibiotic is due to the modulation of the host response since the low-dose formulations have lost their antimicrobial activity [58]. Tetracyclines are found to inhibit MMP activity by cationic binding proteins. The use of this drug in association with mechanical periodontal therapy has been widely accepted. In adults with periodontitis, low-dose doxycycline is currently used as an adjuvant therapy to inhibit MMP activity since it significantly reduces the severity of periodontal disease, including alveolar bone loss [58].

Chemically modified tetracyclines (CMTs) lack the 4-dimethylamino group of TCs, which exerts antibacterial activity [65]. Several CMTs with a range of potency and specificity for MMP inhibition have been developed [65,71,72,73]. CMTs-3 and -8 are the most potent inhibitors of MMPs, especially collagenases, and CMT-3 is the only CMT with demonstrated potency against MMP-1 [66,72].

Different MMPs are also found to be inhibited by chlorhexidine (CHX). CHX is a biguanide chemical substance that provides effective antiseptic effects and is used in the control of plaque and reduction of gingival inflammation [74]. Several authors have shown that chlorhexidine directly inhibits MMP-2, MMP-8, and MMP-9, probably through a chelating mechanism [75,76,77,78,79]. It is proven that CHX dose-dependently inhibits the collagenolytic activity of MMP-8 released by phorbol myristate acetate (PMA)-triggered human polymorphonuclear leukocytes [75].

Bisphosphonates (BPs), pyrophosphate analogs with high affinities for hydroxyapatite crystals, have been used to treat conditions involving increased bone resorption, such as Paget’s disease and osteoporosis [80]. BPs inhibit the dissolution and formation of calcium phosphate crystals, impairing calcification, but their interference with bone resorption is mediated primarily by cellular mechanisms [80]. The most recent generation of BPs, including zoledronate, has been shown to effectively inhibit bone resorption without extensively affecting mineralization [80]. The results of some studies have suggested that BPs do not affect MMP synthesis in cultured carcinoma cells [81] and that they can chelate calcium without affecting osteoclasts or osteoblasts [82], but increasing evidence has indicated that BPs downregulate and inhibit MMPs via chelation [83,84,85].

A recent review stated that adjunctive bisphosphonates in periodontal disease appear to be effective in improving periodontal inflammatory parameters, but several factors such as dosage of drug, frequency of application, and route of administration need to be taken into consideration [86].

Many other molecules broadly inhibit MMP activity. For example, hydroxamate-based inhibitors with structures that mimic collagen, such as batimastat and marimastat, chelate zinc at active sites [25]. However, most newly developed MMP inhibitors allow only selected MMP targeting; synthetic cyclic peptide CTT (HWGFTLC), for example, appears to be gelatinase-specific [87].

Regarding bisphosphonates, due to the antiresorptive properties, there is current interest in their possible use as an adjunctive therapeutic agent for periodontal disease. However, there is still no evidence that bisphosphonates can modify rates and severity of periodontal disease.

## 5. Conclusions

Improved tooth retention, population growth trends, and risk factor modifications have increased the socioeconomic burden of periodontal disease. It has been shown that MMPs can be considered a risk factor for periodontal disease, and measurements of MMP levels may be useful as a biomarker for early detection of periodontitis and as a tool to assess prognostic follow-up.

There seems to be a significant increase in MMP expression and activity in chronic periodontitis, especially MMP-1, MMP-3, MMP-7, MMP-8, and MMP-9, with minor contributions from MMP-13 and MMP-14. Detection of MMPs could, therefore, be useful in periodontal disease prevention. The application of targeted therapy toward MMPs should be considered as a useful addition to periodontal nonsurgical therapy since it could permit the control of periodontal disease and peri-implantitis through an enzymatic modulation, in addition to bacterial control, thus, finally improving oral health globally.

Moreover, the use of certain drugs such as MMP inhibitors could represent an interesting and innovative therapy against MMP effects on periodontal tissues.

## Figures and Tables

**Table 1:** Human matrix metalloproteinases (MMPs) [12].

MMP	Other Names	Group	Substrates in Vitro
MMP-1	Collagenase-1, Interstitial collagenase, Fibroblast collagenase	Collagenases	Type I, II, III, VII, VIII, X, XI collagens, gelatin, fibronectin, laminin, tenascin, a2-macroglobulin, IL-1b, pro-TNF-a, pro-MMP-1, -2, -9
MMP-2	Gelatinase A, 72 kDa gelatinase/type IV collagenase	Gelatinases	Type I, II, III, IV, V, VII, X, XI collagens, gelatin, laminin, elastin, fibronectin, a2-macroglobulin, IL-1b, pro-TNF-a, latent TGF-b, pro-MMP-1, -2, -9, -13
MMP-3	Stromelysin-1, Transin, Proteoglycanase, Collagenase activating protein (CAP)	Stromelysins	Type III, IV, V, VII, IX, X, XI collagens, collagen telopeptides, gelatin, elastin, fibronectin, laminin, aggrecan, decorin, perlecan, versican, a2-macroglobulin, IL-1b, pro-TNF-a, fibrinogen, pro-MMP-1, -3, -7, -8, -9, -13
MMP-7	Matrilysin-1, Putative Metalloprotease (PUMP-1), Matrin	Matrilysins	Type I, IV collagens, gelatin, elastin, fibronectin, laminin, aggrecan, a2-macroglobulin, pro-TNF-a, pro-MMP-1, -2, -7, -9
MMP-8	Collagenase-2, Neutrophil collagenase	Collagenases	Type I, II, III collagens, aggrecan, fibrinogen, a2-macroglobulin, bradykinin
MMP-9	Gelatinase B, 92 kDa gelatinase/type IV collagenase, type V collagenase	Gelatinases	Type IV, V, XI, XIV collagens, gelatin, elastin, laminin, aggrecan, a2-macroglobulin, IL-1b, pro-TNF-a
MMP-10	Stromelysin-2, Transin-2	Stromelysins	Type III, IV, V, VII, IX, X, XI collagens, collagen telopeptides, gelatin, elastin, fibronectin, laminin, aggrecan, decorin, perlecan, versican, a2-macroglobulin, IL-1b, pro-TNF-a, fibrinogen, pro-MMP-1, -3, -7, -8, -9, -13
MMP-11	Stromelysin-3	Stromelysins	Type IV collagen, gelatin, fibronectin, a2-proteinase inhibitor
MMP-12	Macrophage elastase, Metalloelastase	Other MMPs	Type I, IV, V collagens, elastin, gelatin, fibronectin, laminin, aggrecan, a2-macroglobulin, pro-TNF-a, fibrinogen
MMP-13	Collagenase-3	Collagenases	Type I, II, III, IV, VI, IX, X, XIV collagens, collagen telopeptides, gelatin, fibronectin, tenascin-C, aggrecan, fibrinogen, a2-macroglobulin, pro-MMP-9
MMP-14	MT1-MMP	MT-MMPs	Type I, II, III collagens, gelatin, fibronectin, laminin, aggrecan, a2-macroglobulin, pro-TNF-a, fibrinogen, pro-MMP-2, -13, -20
MMP-15	MT2-MMP	MT-MMPs	Fibronectin, tenascin, laminin, aggrecan, pro-TNF-a, pro-MMP-2
MMP-16	MT3-MMP	MT-MMPs	Type III collagen, gelatin, fibronectin, laminin, a2-macroglobulin, pro-MMP-2
MMP-17	MT4-MMP	MT-MMPs	Gelatin, fibrinogen, fibrin, pro-TNF-a
MMP-19	Matrix metalloproteinase RASI-1	Other MMPs	Type IV collagen, gelatin, laminin, fibronectin, fibrinogen, fibrin
MMP-20	Enamelysin	Other MMPs	Amelogenin, type IV collagen, aggrecan, fibronectin, laminin, tenascin-C
MMP-21	-	Other MMPs	a1-antitrypsin
MMP-23	Cysteine array (CA)-MMP	Other MMPs	Gelatin
MMP-24	MT5-MMP	MT-MMPs	Fibronectin, gelatin, chondroitin sulfate proteoglycan, pro-MMP-2
MMP-25	MT6-MMP, Leukolysin	MT-MMPs	Type IV collagen, gelatin, fibronectin, fibrinogen, fibrin, pro-MMP-2
MMP-26	Matrilysin-2	Matrilysins	Gelatin, fibronectin, a2-macroglobulin, fibrinogen, pro-MMP-9
MMP-27	-	Other MMPs	-
MMP-28	Epilysin	Other MMPs	Casein

TNF = Tumor Necrosis Factor; TGF = Transforming Growth Factor.

**Table 2:** Selected studies about MMPs involved in periodontal tissue degradation.

Author (Year)	MMP Involved Into Periodontal Tissues Degradation
Choi D.H. et al. (2004) [27]	MMP-8, MMP-9
Beklen A. et al. (2006) [28]	MMP-8, MMP-9
Soder B. et al. (2006) [29]	MMP-8, MMP-9
Kumar M.S. et al. (2006) [30]	MMP-8, MMP-9
Marcaccini A.M. et al. (2010) [31]	MMP-1, MMP-13
Kraft-Naumarker M. et al. (2012) [32]	MMP-8
Rai B. et al. (2008) [34]	MMP-8, MMP-9
Xu L. et al. (2008) [35]	MMP-8
Schure R. et al. (2013) [36]	MMP-3, MMP-7, MMP-8
Gursoy U.K. et al. (2010) [37]	MMP-8
Eminigil G. et al. (2006) [39]	MMP-7
Eminigil G. et al. (2006) [40]	MMP-25, MMP-26
Ramseier C.A. et al. (2009) [41]	MMP-8, MMP-9
Hernandez Rios M. et al. (2009) [42]	MMP-9, MMP-13
Alpagot T. et al. (2001) [43]	MMP-3
Marcaccini A.M. et al. (2009) [44]	MMP-3, MMP-8, MMP-9
Kinane D.F. et al. (2003) [45]	MMP-8
Mantyla P. et al. (2006) [46]	MMP-8
Pozo P. et al. (2005) [47]	MMP-8, MMP-9
Mantyla P. et al. (2003) [48]	MMP-8
